# Supplementary material for: Patterns of Intron Gain and Loss in Fungi
Source: PLoS Biol. 2004 Nov 30;2(12):e422. doi: 10.1371/journal.pbio.0020422 (PMC532390; doi:10.1371/journal.pbio.0020422)
Supplement: Table S1 — Also available at http://genes.mit.edu/NielsenEtAl/. (4.3 MB ZIP). [file pbio.0020422.st001.zip › NielsenEtAl/html/949.html]

AN1016.1.NCU05206.1.MG01818.1.FG09614.1


```
 CLUSTAL W (1.82) Multiple Sequence Alignments - Introns Inserted


Sequence 1: MG01818.1	356 aa
Sequence 2: FG09614.1	355 aa
Sequence 3: NCU05206.1	356 aa
Sequence 4: AN1016.1	356 aa
Alignment Length: 358 aa
Number Identitical Residues: 262 aa
Alignment Score (without introns) 10845


MG01818.1 	MMGACMSASSE-ESEQKKRSQKIDKDLEEDSKRLRRECKILLLG1SGESGKSTIVKQMKI
NCU05206.1	-MGACMSKNDE-ETEQKKRSQKIDRDLEEDSKKLRKECKILLLG1SGESGKSTIVKQMKI
FG09614.1 	-MGACMSSSNE-EVDQKKKSQAIDKILEEDSKRLRKECKILLLG1SGESGKSTIVKQMKI
AN1016.1  	-MGSCFSSESAGDVEQKKRSQAIDRKLEEDSRRLRRECKILLLG1SGESGKSTIVKQMKI
          	 **:*:* .. .: :***:** **: *****::**:******** ***************

MG01818.1 	IHLKGYSDDELYNYRPTVFKNLIECAKAVISAMRQFDIEPQLEGNKELCDYLLDYMVESG
NCU05206.1	IHLKGYSDEELTNYRPTVYKNLLECAKAVVNAMHQFDIQPADPSLRPYVEFLQDYNMEGC
FG09614.1 	IHLKGYSEDELFNYRPTVFKNLVECAKAVIMAMQQFDIEPQNEENKAHAEFLLEYQAESG
AN1016.1  	IHQNGYTVEELALYRLTVYKNLLECAKALIGAYHQFNLEPTSQKVRDNIEFLANYNIDPD
          	** :**: :**  ** **:***:*****:: * :**:::*     :   ::* :*  :  

MG01818.1 	PQ-AEIDPKVGQAVQSIWNDPAREQLMDRQTEFYLMDSAEY2FFQEVLRIVSPDYLPNEM
NCU05206.1	PPGQSIDPKVGTAIQALWNDPAKEQLMERQTEFYLMDSAEY2FFTEVMRIVAEDYRPNEM
FG09614.1 	PQ-AHIDAKVGAAVQALWNDPAKDLLMEHQTEFYLMDSAEY2FFQEAMRIVATDYLPNEM
AN1016.1  	PN-IPLDPAVGDAITYIWNDPCTSTALERQNEFYLMDSAPY2FFEEAKRITSPDYIPNVN
          	*    :*. ** *:  :****. .  :::*.******** * ** *. **.: ** **  

MG01818.1 	DVLRARTKTTGIYETRFQMGQLSIH2MFDVGGQRSERKKWIHCFENVTSIIFCVALSEYD
NCU05206.1	DVLRARTKTTGIYETRFKMGQLSIH2MFDVGGQRSERKKWIHCFENVTSIIFCVALSEYD
FG09614.1 	DVLRARTKTTGIYETRFQMGQLSIH2MFDVGGQRSERKKWIHCFENVTSIIFCVALSEYD
AN1016.1  	DVLRARTKTTGIYETRFTMGQLSIH2MFDVGGQRSERKKWIHCFENVTSIIFCVALSEYD
          	***************** ******* **********************************

MG01818.1 	QVLLEESSQ0NRMMESLLLFDSVVNSRWFMRTSIILFLNKVDIFKQKLGRSPLGNYFPDY
NCU05206.1	QVLLEESSQ0NRMMESLLLFDSVVNSRWFMRTSIILFLNKVDIFKQKLGRSPLGNYFPDY
FG09614.1 	QVLLEESSQ0NRMMESLLLFDSVVNSRWFMRTSIILFLNKVDIFKQKLSRSPLGNYFPDY
AN1016.1  	QVLLEEGNQ0NRMMESLVLFDSVVNSRWFMRTSIILFLNKVDLFRQKLPRSPLSNYFPDY
          	******..* *******:************************:*:*** ****.******

MG01818.1 	SGGNDVNKAAKYLLWRFNQVNRAHLNLYPH2LTQATDTSNIRLVFAAVKETILNNALKDS
NCU05206.1	SGGNDVNKAAKYLLWRFNQVNRAHLNLYPH2LTQATDTSNIRLVFAAVKETILNNALKDS
FG09614.1 	SGGNDVNKAAKYLLWRFNQVNRAHLNLYPH2LTQATDTSNIRLVFAAVKETILNNALKDS
AN1016.1  	SGGNDVNRAAKYLLWRFNQVNRAHLNLYPH2LTQATDTTNIRLVFAAVKETILQNALKDS
          	*******:********************** *******:**************:******

MG01818.1 	GIL
NCU05206.1	GIL
FG09614.1 	GIL
AN1016.1  	GIL
          	***
```
